# Cerebrospinal Fluid Aβ43 Is Reduced in Early-Onset Compared to Late-Onset Alzheimer’s Disease, But Has Similar Diagnostic Accuracy to Aβ42

**DOI:** 10.3389/fnagi.2017.00210

**Published:** 2017-06-28

**Authors:** Camilla Lauridsen, Sigrid B. Sando, Ina Møller, Guro Berge, Precious K. Pomary, Gøril R. Grøntvedt, Øyvind Salvesen, Geir Bråthen, Linda R. White

**Affiliations:** ^1^Department of Neuromedicine and Movement Science, Faculty of Medicine and Health Sciences, Norwegian University of Science and TechnologyTrondheim, Norway; ^2^Department of Neurology, Trondheim University HospitalTrondheim, Norway; ^3^Unit for Applied Clinical Research, Faculty of Medicine and Health Sciences, Norwegian University of Science and TechnologyTrondheim, Norway

**Keywords:** early-onset Alzheimer’s disease, biomarkers, tau, YKL-40, neurofilament light, glial fibrillary acidic protein, progranulin

## Abstract

**Background:** Amyloid beta 1–43 (Aβ43) may be a useful additional biomarker for diagnosing Alzheimer’s disease (AD). We have investigated cerebrospinal fluid (CSF) levels of Aβ43 in patients with early-onset AD in contrast to levels in late-onset AD. For comparison, in addition to the ‘core’ biomarkers, several other analytes were also determined [YKL-40, neurofilament light (NF-L), glial fibrillary acidic protein (GFAP), and progranulin].

**Material and Methods:** Cerebrospinal fluid samples were obtained from patients with early-onset AD (age ≤ 62, *n* = 66), late-onset AD (age ≥ 68, *n* = 25), and groups of cognitively intact individuals (age ≤ 62, *n* = 41, age ≥ 68, *n* = 39). Core CSF AD biomarkers [amyloid beta 1–42 (Aβ42), total tau, phosphorylated tau] were analyzed, as well as levels of Aβ43 and other analytes, using commercially available enzyme-linked immunosorbent assays.

**Results:** Cerebrospinal fluid Aβ43 was significantly reduced in early-onset AD compared to late-onset AD (14.8 ± 7.3 vs. 21.8 ± 9.4 pg/ml, respectively), whereas the levels of Aβ42 in the two AD groups were not significantly different (474.9 ± 142.0 vs. 539.6 ± 159.9 pg/ml, respectively). Aβ43 and all core biomarkers were significantly altered in patients with AD compared to corresponding controls. NF-L was significantly increased in early-onset AD compared to younger controls, an effect not found between the older groups. Relationships between the Aβ peptides and tau proteins, YKL-40, NF-L, GFAP and progranulin were also investigated without finding marked associations. However, age-associated increases in levels of tau proteins, YKL-40, NF-L and GFAP were found with respect to age in healthy controls. Results for these other analytes were similar to previously published data. Aβ43 did not improve diagnostic accuracy in either AD group compared to Aβ42. Discussion: Cerebrospinal fluid Aβ43, but not Aβ42 levels, varied significantly with age in patients with AD. If CSF levels of Aβ peptides reflect amyloid deposition in brain, the possibility arises that there is a difference between Aβ43 and Aβ42 deposition in younger compared to older brain. However, the level of Aβ43 in CSF shows no improvement over Aβ42 regarding diagnostic accuracy.

## Introduction

Alzheimer’s disease (AD) is often separated according to age, whereby onset prior to age 65 years is considered to be early-onset AD, while onset from an age of 65 years (which is much more common) is termed late-onset AD. Although the pathological burden of amyloid plaques and neurofibrillary tangles has been shown to be greater in early-onset AD than in patients with late-onset AD (Ho et al., 2002; Marshall et al., 2007), imaging studies have indicated the global burden to be similar between the two groups (Rabinovici et al., 2010), though sometimes with variation in regional anatomical distribution of amyloid (Ossenkoppele et al., 2012; Cho et al., 2013). Such putative differences in the distribution of amyloid pathology have not been found to alter levels of the core cerebrospinal fluid (CSF) biomarkers for AD; amyloid beta 1–42 (Aβ42), total tau (t-tau) and phosphorylated tau (p-tau) protein, in early- compared to late-onset AD (Bouwman et al., 2009; Chiaravalloti et al., 2016). Additionally, several studies have shown no correlation between the core biomarkers and age in AD patients (Bouwman et al., 2009; Mattsson et al., 2009; Popp et al., 2010).

However, healthy individuals display increased AD pathology with increasing age (Savva et al., 2009). Older control individuals have been found to have decreased CSF Aβ42 levels compared to younger controls (Bouwman et al., 2009), and the level was negatively correlated with age (Popp et al., 2010). Conversely, CSF t-tau and p-tau correlate positively with age in healthy elderly individuals (Blomberg et al., 2001; Glodzik-Sobanska et al., 2009; Jaworski et al., 2009; Alcolea et al., 2015).

Amyloid beta 1–43 (Aβ43), compared to Aβ42, has an additional threonine at the C-terminal through an alternative γ-secretase cleavage of amyloid precursor protein (APP), and is generally considered likely to be even more aggregation-prone than Aβ42 (Jarrett et al., 1993; Saito et al., 2011; Conicella and Fawzi, 2014), though recent kinetic experiments *in vitro* dispute this (Chemuru et al., 2016). Aβ43 has been hypothesized to play a role in AD pathogenesis despite its low concentration in human brain tissue, and found to be frequent in both neuritic and diffuse extracellular plaques in both familial and sporadic AD (Welander et al., 2009; Keller et al., 2010; Sandebring et al., 2013). In an APP-expressing transgenic mouse model, Aβ43 has been found to be the first amyloid peptide to deposit in brain (Zou et al., 2013), perhaps seeding subsequent Aβ42 deposition (Conicella and Fawzi, 2014). Moreover, knock-in mice bearing the pathogenic presenilin-1 R278I mutation demonstrated overproduction of Aβ43, impaired short-term memory and acceleration of amyloid-β pathology (Saito et al., 2011). Aβ43 correlates positively with age in patients with AD (Bruggink et al., 2013), and correlates closely with CSF Aβ42 both in patients and control individuals (Bruggink et al., 2013; Lauridsen et al., 2016). As far as we know, no study as yet has compared CSF levels of Aβ43 in early-onset AD with late-onset AD.

We have therefore investigated Aβ43 and Aβ42 in CSF from well-characterized cohorts of patients with early- and late-onset AD. The cut-off between these subtypes of AD has been accepted as 65 years of age, but in the present study we excluded patients with age at onset in the 5-year period 63–67 years to highlight potential age differences. Thus only patients with early-onset AD ≤ 62 years of age, or patients with late-onset AD who were aged ≥68 years were included.

In addition to the Aβ species in CSF from these two subgroups of patients with AD and corresponding control groups, levels of t-tau and p-tau were also determined. For further comparison, levels of several other analytes that have been investigated with respect to AD, and to age, were also assessed. These included two other cytoskeletal intermediate filaments; neurofilament light (NF-L) (Petzold et al., 2007; Vagberg et al., 2015; Olsson et al., 2016) and glial fibrillary acidic protein (GFAP) (Vagberg et al., 2015; Wennstrom et al., 2015), found, respectively, in neurons and glia, YKL-40 (also known as chitinase 3-like protein 1), a protease secreted mainly by astrocytes and considered a marker for gliosis and neuroinflammation (Craig-Schapiro et al., 2010), and progranulin, a growth factor believed to have anti-inflammatory and neuroprotective abilities (Jing et al., 2016).

## Materials and Methods

### Subjects

Study patients were ethnic Norwegians referred to the Department of Neurology, St. Olav’s Hospital (Trondheim University Hospital) by general practitioners, and diagnosed by a neurologist. Some patients were initially diagnosed with amnestic mild cognitive impairment (aMCI, *n* = 14) according to the International Working Group on Mild Cognitive Impairment criteria (Winblad et al., 2004), but all later developed AD within the next 2 years. Patients with AD were diagnosed according to the NINCDS-ADRDA criteria (McKhann et al., 1984), final total *n* = 91, whereof 66 were aged ≤62 years at onset (early-onset AD) and 25 were aged ≥68 years at onset of symptoms (late-onset AD).

As controls, CSF samples were obtained either from non-demented elderly volunteers (*n* = 35) recruited from societies for retired people or caregivers not genetically related to the patient, or from samples stored in the Neurological Research Biobank at the hospital (*n* = 45). These latter individuals had been referred to the clinic for suspected neurological conditions, but none was subsequently found. Of the total 80 control individuals, 41 were aged ≤62 years, and 39 were aged ≥68 years. For the control groups, CSF cell count, glucose and protein were within standard physiological limits.

The neurological examination performed on most study participants included the Mini Mental State Examination (MMSE) (Folstein et al., 1975). MMSE was performed on all patients, but for many control individuals there had been no reason to carry out an MMSE during the clinical work-up, and where MMSE was available, the minimum score was 28. For the same reason, *APOE* genotype was not available for most younger controls. The demographic data are shown in **Table 1**.

**Table 1:** Demographic and CSF biochemical data.

	Controls age ≤ 62	Early-onset AD age ≤ 62	Controls age ≥ 68	Late-onset AD age ≥ 68
Total n	41	66	39	25
Gender (female/male)	21/20	37/29	23/16	15/10
Age at inclusion (y)	57 (47–62)	61 (51–67)^A^∗∗^^	71 (68–84)	76 (71–84)^B^∗^^
Age at onset (y)	N/A	58 (47–62)	N/A	73 (68–82)
Duration (y)	N/A	3 (1–11)	N/A	2 (1–5)
MMSE score	29 (28–30)	24 (10–30)^A^∗∗^^	29 (28–30)	23 (12–29)^B^∗∗^^
	12	63	33	25
*APOE* genotype (% with an 𝜀4 allele, total n genotyped)	37.5	74.2^#^	45.2	72.2^#^
	8	62	31	18
Aβ43 (pg/ml)	38.0 ± 14.6	14.8 ± 7.3^A^∗∗^C^∗∗^^	45.8 ± 13.7	21.8 ± 9.4^B^∗∗^^
	37	50	23	24
Aβ42 (pg/ml)	844.9 ± 220.9	474.9 ± 142.0^A^∗∗^^	967.5 ± 247.2	539.6 ± 159.9^B^∗∗^^
	31	64	36	25
t-tau (pg/ml)	246.5 ± 99.5^B^∗^^	767.8 ± 485.5^A^∗∗^^	348.0 ± 166.8	646.9 ± 418.6^B^∗^^
	32	64	37	25
p-tau (pg/ml)	42.6 ± 18.1^B^∗∗^^	98.1 ± 39.5^A^∗∗^^	60.8 ± 20.8	98.8 ± 51.4^B^∗^^
	32	64	37	25
YKL-40 (ng/ml)	139.1 ± 55.6^B^∗∗^^	206.0 ± 97.4^C^∗^^	237.3 ± 73.2	287.3 ± 109.8
	38	45	34	23
NF-L (pg/ml)	567.2 ± 190.0^B^∗∗^^	1497.8 ± 814.5^A^∗∗^^	1381.4 ± 1419.3	1882.0 ± 2122.2
	41	51	39	24
GFAP (pg/ml)	1227.5 ± 475.3^B^∗^^	1889.8 ± 1072.1^A^∗^^	1786.8 ± 608.3	2210.8 ± 903.1
	13	14	25	21
Progranulin (pg/ml)	4844.2 ± 1349.8	4855.3 ± 1395.5	5358.8 ± 977.1	5403.6 ± 1064.9
	37	38	8	21

*Demographic data are given as the median (range) for continuous variables, CSF biochemical data are given as the mean ± SD, with the number of analyses. Statistical analysis was performed with pairwise group comparisons of log-transformed analyte levels between controls and AD patients aged ≤62 years (age-adjusted), and between controls and AD patients aged ≥68 years (age-adjusted), as well as between younger and older groups of controls, and younger and older groups of patients with AD. ^A^Significantly different to younger controls, ^B^significantly different to older controls, ^C^significantly different to late-onset AD patients. ^∗^*p* < 0.01, ^∗∗^*p* < 0.001. ^#^Increased frequency of the *APOE* 𝜀4 allele in patient compared to control groups (*p* = 0.001). AD, Alzheimer’s disease; N/A, not applicable; MMSE, Mini Mental State Examination; APOE, apolipoprotein E; y, years; Aβ, amyloid beta; t-tau, total tau; p-tau, phosphorylated tau; NF-L, neurofilament light; GFAP, glial fibrillary acidic protein.*

### Sampling of CSF

Cerebrospinal fluid was collected with patients lying on their side, and lumbar puncture carried out at the level L4/L5 or L5/S1. The first 2.5 mL CSF was used for routine clinical investigation. Aliquots of CSF were collected directly into polypropylene cryovials (Corning) immersed in ice-water. No samples used in this study were contaminated by blood, and so were not centrifuged. All samples were frozen within 30 min of lumbar puncture and stored at -80°C until analysis. Ten samples were thawed and then frozen again before core biomarkers were analyzed. One freeze-thaw cycle has previously been shown to not significantly affect core biomarker results (Le Bastard et al., 2015).

### ELISA Assays

Cerebrospinal fluid samples were analyzed using ELISA monoplex kits according to the manufacturers’ instructions [Aβ43 (IBL), Aβ42 (Innogenetics), t-tau (Innogenetics), p-tau (Innogenetics), NF-L (UmanDiagnostics), YKL-40 (Bio-Techne, CSF diluted 1:400), GFAP (BioVendor) and progranulin (Adipogen Life Sciences, CSF diluted 1:15)]. Samples were thawed in ice-water prior to analysis, and all samples were analyzed in duplicate. Cross-reactivity for Aβ42 in the Aβ43 ELISA was given as <1%. Although this would contribute slightly to measurements for Aβ43, it would be a constant for both control and patient groups. Aβ43 was reported to have 50× less affinity than Aβ42 for the antibodies in the Aβ42 kit.

### Statistical Analysis

Statistical analyses were carried out using SPSS version 24 (IBM) and Stata version 13.1. Due to multiple testing, *p*-values < 0.01 were considered statistically significant. Distribution of gender between groups and the distribution of the *APOE* 𝜀4 allele between groups were assessed with Pearson’s χ^2^ (chi-square) test. Differences in age at inclusion between patients with early- or late-onset of AD and respective control groups, as well as for MMSE scores and duration of disease, were assessed with the independent samples Mann–Whitney U-test for pairwise comparisons of groups. CSF analyte levels were log-transformed to approximate a normal distribution. Analyte levels were compared for younger and older participants within control and AD patient groups using *t*-tests for independent samples. However, when comparing analyte levels between controls and AD patients it was necessary to adjust for age because patients were significantly older than controls in both age groups. Analyte levels for the group of younger controls were therefore compared with those of early-onset AD patients, and older controls with those of late-onset AD patients using linear regression and adjusting for age at inclusion. Correlations between analytes, or between analytes and age at inclusion, were calculated with Pearson’s r. Associations are only tentative as both type 1 and type 2 errors can occur even employing a significance level of *p* < 0.01 as in the present study. Patterns as a whole have been considered more informative than individual correlations. To investigate potential differences in diagnostic accuracy, receiver operating characteristic (ROC) curves were made for Aβ43 and Aβ42, and the area under each ROC curve (AUC) was calculated. Youden’s index was found to determine where the sum of sensitivity and specificity was maximized. AUC was compared between Aβ43 and Aβ42 for controls and AD patients in corresponding groups [DeLong method (DeLong et al., 1988)]. Levels of analyte ratios were compared between groups, but overall did not separate groups more clearly than single analytes, and are therefore not considered further. Ratio data are given in Supplementary Table S1.

### Ethics Statement

The study was conducted according to the Helsinki Declaration. Written, informed consent was obtained from all patients or suitable proxies, and from all control individuals. The Neurological Research Biobank has been licensed by the Norwegian Directorate for Health Affairs, and the research was approved by the Regional Committee for Medical Research Ethics (approval 2010/226 REK Midt, 2013/467 REK Midt, 2013/150 REK Sør-Øst).

## Results

When comparing participant groups, there were no significant differences in the distribution of gender. The median age at inclusion in both younger and older control groups was significantly lower than for corresponding patient age groups. There was no significant difference in the duration of disease between the two patient groups. No significant differences were found in MMSE scores between individuals in the respective control or patient groups. Both patient groups had significantly lower median MMSE scores than their respective control group. There was increased frequency of the *APOE* 𝜀4 allele in combined patient compared to combined control groups (*p* = 0.001) (**Table 1**).

**FIGURE 1:**
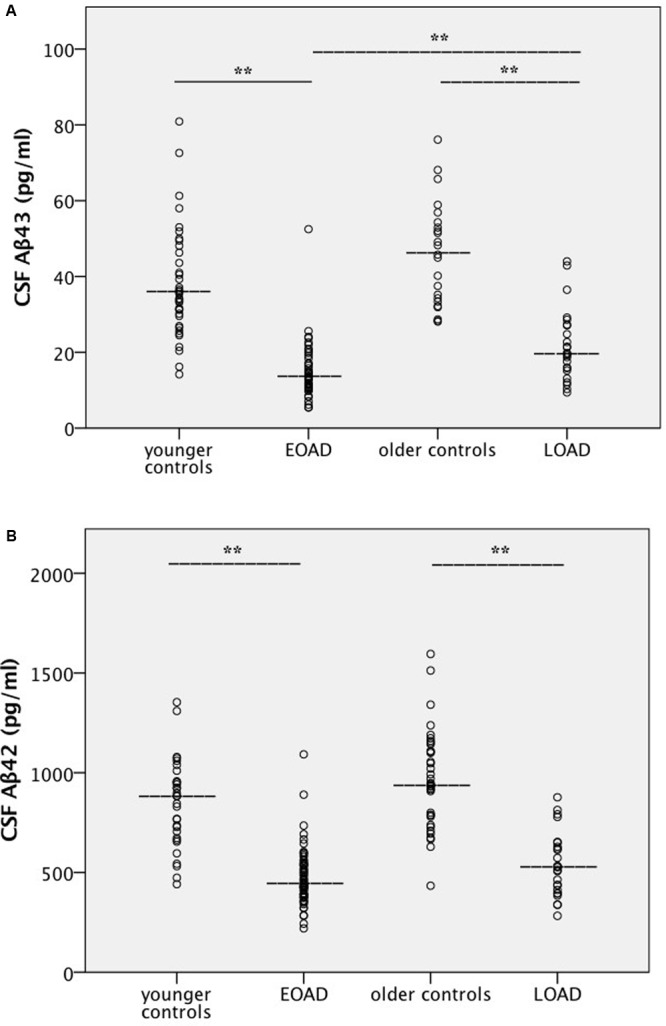
**(A,B)** Amyloid levels in cerebrospinal fluid. Scatter plots for all four participant groups with median lines added for each group. Values for the mean ± 1 SD are given in **Table 1**. Statistical analysis was performed with pairwise group comparisons of log-transformed analyte levels between controls and AD patients aged ≤62 years (age-adjusted), and between controls and AD patients aged ≥68 years (age-adjusted), as well as between younger and older groups of controls, and younger and older groups of patients with AD. **(A)** Aβ43, **(B)** Aβ42. ^∗∗^Significantly different at the *p* < 0.001 level. AD, Alzheimer’s disease; Aβ, amyloid beta; EOAD, early-onset AD; LOAD, late-onset AD.

Cerebrospinal fluid levels of the various analytes are shown in **Table 1**, and scatter plots for amyloid peptides are shown in **Figure 1** and in Supplementary Figures S1A–F for the other analytes. Additionally, correlations between CSF levels of Aβ peptides and other analytes, and between Aβ peptide levels and age were calculated. CSF Aβ43 was significantly decreased in patients with early-onset AD compared to late-onset AD, but no significant difference was found between the two patient groups for Aβ42. There were highly significant reductions in the levels of both Aβ43 and Aβ42 in CSF of patients with AD compared to controls. No significant differences in levels of Aβ43 or Aβ42 were found between the two control groups. Both CSF Aβ43 and Aβ42 were excellent at separating corresponding controls from patients in the AD groups, with AUCs of 0.93 or better and no significant difference in AUCs between Aβ43 and Aβ42 (**Table 2**). Aβ43 and Aβ42 correlated significantly with each other in all four participant groups (*r* = 0.58–0.85, *p* ≤ 0.006).

**Table 2:** Diagnostic accuracy of β-amyloids for the separation of controls and patients.

	Age ≤ 62 years	Age ≥ 68 years
CSF Aβ43	AUC: 0.96 Sensitivity: 96% Specificity: 89%	AUC: 0.94 Sensitivity: 79% Specificity: 100%
CSF Aβ42	AUC: 0.93 Sensitivity: 92% Specificity: 84%	AUC: 0.93 Sensitivity: 84% Specificity: 94%

*Aβ, amyloid beta; AUC, area under the receiver operating characteristic curve.*

A significant positive association between Aβ42 and age at inclusion was found in younger controls (*r* = 0.55, *p* = 0.001) and in early-onset AD (*r* = 0.38, *p* = 0.002). For older controls a trend was found for a negative correlation (*r* = -0.42, *p* = 0.012), but this was lost in late-onset AD. For Aβ43, a positive correlation with age at inclusion was found in the early-onset AD group (*r* = 0.43, *p* = 0.002), but the association was not significant in the other three participant groups.

Results for t-tau and p-tau were similar in nature, and both correlated with each other in all groups (*r* = 0.76–0.93, all *p* < 0.001). Their levels were significantly increased in patients compared to the corresponding control group, but there was no difference between patients with early- or late-onset AD. However, the older control group had significantly higher levels of the tau species compared to younger controls (**Table 1**). Associations between tau proteins and Aβ peptides were found only in younger controls (*r* = 0.43, *p* = 0.016 to *r* = 0.52, *p* = 0.003), not older controls or either AD group.

YKL-40 was not significantly increased in patients compared to the respective control group. However, a significant increase was found between early- and late-onset AD, as well as between younger and older controls. There was a pattern for a relationship between the Aβ peptides and YKL-40 in younger controls and early-onset AD, but correlation coefficients were low (all *r* = 0.33–0.44, *p* < 0.05 except for Aβ43 and YKL-40 in early-onset AD, *p* = 0.003).

A highly significant increase in the level of NF-L was found in early-onset AD compared to younger controls, but this difference was lost between late-onset AD and older controls. There was no difference between the two groups of patients, but older controls had significantly higher levels of NF-L compared to younger controls. Levels of GFAP in patients with early-onset AD were significantly higher than in younger controls. Older controls also had significantly increased levels compared to the younger controls, but no significant differences between the patient groups were found. No significant group differences in progranulin levels were found in this material.

## Discussion

The most interesting result in this study is that the reduction in CSF levels of Aβ43 was more marked in early-onset compared to late-onset AD, and therefore seems to be age-related. This difference was not found for Aβ42. As expected, there was a clear and highly significant reduction in the concentration of both Aβ43 and Aβ42 in the CSF of the patient groups compared to corresponding controls. However, the data do not suggest that Aβ43 has better diagnostic accuracy for AD than Aβ42.

The increased deposition of parenchymal Aβ species in the AD brain has been suggested as the reason for the reduced amounts of Aβ peptides measured in CSF (usually Aβ42), based on the idea that less may be available for passage over the brain-CSF barrier (Fagan et al., 2006). Recent results from imaging studies showed that although CSF Aβ43 is strongly associated with cerebral amyloid deposits, even at early stages of clinical cognitive impairment (subjective cognitive decline and MCI), there were no relative differences in deposition between Aβ42 and Aβ43. Aβ43 therefore provided no diagnostic improvement over the established marker Aβ42 (Almdahl et al., 2017). Also in the present study comparing early- and late-onset AD versus the corresponding control group, no improvement to diagnostic accuracy was found for Aβ43 compared to Aβ42.

It is not immediately obvious why Aβ43 would be reduced more in early-onset than late-onset AD, other than that there is an age difference between the patient groups. However, two studies comparing amyloid imaging in early- and late-onset AD report regional (though not identical) differences in fibrillar amyloid deposition (Ossenkoppele et al., 2012; Cho et al., 2013). It is therefore possible there are age-related differences in the topographical deposition of Aβ peptides, but whether this would produce differences in CSF concentrations of the peptides in early-onset compared to late-onset AD remains unclear. We did not find a similar reduction for CSF Aβ42 in early-onset AD, and this result is very similar to previously published data (Gronning et al., 2012).

In healthy individuals, several studies have found little or no correlation between age and Aβ42 levels in CSF (Hansson et al., 2006; Bouwman et al., 2009; Mattsson et al., 2009; Popp et al., 2010). Similarly in patients with AD, a number of articles report no correlation between age and Aβ42 levels (Bouwman et al., 2009; Mattsson et al., 2009; Popp et al., 2010). Our data were similar in this respect. Even though significant correlations between age and Aβ42 in controls and patients were found, none were strong, and there was no pattern. The significance of weak correlations is dependent on the number of samples included and the significance level applied. Given that even strong correlations do not guarantee biological relevance, it can be questioned whether these fairly weak correlations are sufficiently reliable to warrant speculation of underlying physiological changes.

Generally speaking, our results for the core biomarkers in controls and in AD, as well as the association with age, agree broadly with previous studies (Blomberg et al., 2001; Bouwman et al., 2009; Glodzik-Sobanska et al., 2009; Popp et al., 2010; Alcolea et al., 2015; Chiaravalloti et al., 2016; Olsson et al., 2016). Age is also important for other substances analyzed in the present study. In recent years several reports have shown that YKL-40 increases throughout middle-age in cognitively healthy individuals, suggesting that a certain level of neuroinflammation is physiological in normal aging (Alcolea et al., 2015; Sutphen et al., 2015), as well as being an aspect of AD (Wennstrom et al., 2015). The present study agrees with the finding of increased YKL-40 levels with increased age. There was a pattern of positive correlations between Aβ species and YKL-40 in the younger groups. Most of the correlations were rather weak so it is uncertain whether this represents a physiological relationship, but tentatively agrees with previous data indicating that markers of inflammation, including YKL-40, and Aβ42 in normal aging and the early AD pathological process, are related (Alcolea et al., 2015). NF-L is well known to be increased in the CSF of patients with AD (Petzold et al., 2007; Olsson et al., 2016), and the present results are in accordance with this, but only in connection with early-onset AD. No significant difference was found for CSF NF-L between late-onset AD and older controls, which probably reflects the increase of CSF NF-L in normal aging (Vagberg et al., 2015). Similarly, we found an increase in CSF GFAP in patients with early-onset AD compared to younger controls, but again perhaps due to the increase in CSF levels of GFAP with age (Vagberg et al., 2015), this difference was lost between late-onset AD and older controls. The results for the older groups agree with one study (Wennstrom et al., 2015), but not with another study that found increased GFAP levels in AD patients compared to controls (Jesse et al., 2009). No changes in the concentration of progranulin in CSF from patients with AD were found compared to controls, as previously demonstrated (Morenas-Rodriguez et al., 2016).

Taken together, few differences were detected between early- and late-onset AD when analyzing CSF for potential markers of disease, even though the two groups had been clearly defined with respect to a difference in age. When comparing patients and controls, more differences were associated with early-onset rather than late-onset AD, perhaps because both patients and controls tend to suffer more comorbidities with increasing age which can cloud differences between patients with AD and controls. The main strength of the present study was to employ clinically well-defined patient and control cohorts that were large enough to distinguish differences and similarities in Aβ43 and Aβ42. In light of an earlier report (Lauridsen et al., 2016), future studies should probably concentrate on examining CSF Aβ43 and Aβ42 in relation to early stages of the AD process, including amnestic MCI, subjective cognitive decline, and cognitively intact individuals who have a pathological pattern of core biomarkers in CSF, or increased amyloid deposition in brain.

## Author Contributions

CL planned and performed the laboratory work together with IM, PP, and GBe. SS was responsible for clinical aspects together with GG and GBr. CL analyzed the data and ØS advised on statistical analysis. LW was responsible for study design and data collation together with CL and SS. LW, SS and GBr supervised the project. CL and LW wrote the manuscript. All authors contributed to critical revision and finalization of the manuscript.

## Conflict of Interest Statement

The authors declare that the research was conducted in the absence of any commercial or financial relationships that could be construed as a potential conflict of interest.
