# Supplementary material for: Cerebrospinal Fluid Aβ43 Is Reduced in Early-Onset Compared to Late-Onset Alzheimer’s Disease, But Has Similar Diagnostic Accuracy to Aβ42
Source: Front Aging Neurosci. 2017 Jun 28;9:210. doi: 10.3389/fnagi.2017.00210 (PMC5487529; doi:10.3389/fnagi.2017.00210)
Supplement: Supplementary file 1 [file Data_Sheet_1.docx]

**Supplementary Table 1.** Ratios of the analytes in cerebrospinal fluid.

|  | Controls ≤62 | Early-onset AD ≤62 | Controls ≥68 | Late-onset AD ≥68 |
| --- | --- | --- | --- | --- |
| Aβ42 / Aβ43 | 24.0 ± 6.7  27 | 35.6 ± 12.6 **^A, B^**  48 | 22.7 ± 5.5  21 | 26.6 ± 6.0  24 |
| t-tau / Aβ43 | 7.2 ± 2.8  28 | 57.6 ± 31.0 **^A, B^**  48 | 6.9 ± 3.6  22 | 34.3 ± 33.5 **^A^**  24 |
| t-tau / Aβ42 | 0.3 ± 0.1  31 | 1.7 ± 1.3 **^A^**  64 | 0.4 ± 0.4  36 | 1.3 ± 1.1 **^A^**  25 |
| YKL-40 / Aβ43 | 4028 ± 2198  37 | 15863 ± 8478 **^A^**  43 | 4936 ± 1921  21 | 15957 ± 9588 **^A^**  23 |
| YKL-40 / Aβ42 | 164.6 ± 62.3 **^C^**  28 | 460.1 ± 246.7 **^A^**  43 | 272.7 ± 149.7  31 | 595.2 ± 296.3 **^A^**  23 |
| Progranulin / Aβ43 | 142.1 ± 60.9  37 | 410.6 ± 203.9 **^A^**  38 | 122.9 ± 39.9  8 | 310.4 ± 136.2 **^A^**  21 |
| Progranulin / Aβ42 | 6.2 ± 2.3  27 | 11.0 ± 3.0 **^A^**  36 | 6.4 ± 1.9  8 | 11.4 ± 3.6 **^A^**  21 |
| NF-L / GFAP | 0.6 ± 0.4  13 | 0.9 ± 0.6  14 | 0.9 ± 0.7  25 | 0.9 ± 0.6  21 |

Data are given as the mean ± SD, with the number of analyses. Statistical analysis was performed with pairwise group comparisons of log-transformed analyte levels between controls and AD patients aged ≤62 years (age-adjusted), and between controls and AD patients aged ≥68 years (age-adjusted), as well as between younger and older groups of controls, and younger and older groups of patients with AD. **^A^** significantly different to corresponding control group, p≤0.001, **^B^** significantly different to patients with late-onset AD, p≤0.001, **^C^** significantly different to older control group, p<0.001. Abbreviations: AD = Alzheimer’s disease, Aβ = amyloid beta, t-tau = total tau protein, p-tau = phosphorylated tau protein, NF-L = neurofilament light, GFAP = glial fibrillary acidic protein.
