# Supplementary material for: Cerebrospinal Fluid Aβ43 Is Reduced in Early-Onset Compared to Late-Onset Alzheimer’s Disease, But Has Similar Diagnostic Accuracy to Aβ42
Source: Front Aging Neurosci. 2017 Jun 28;9:210. doi: 10.3389/fnagi.2017.00210 (PMC5487529; doi:10.3389/fnagi.2017.00210)

**Supplementary Figure S1A-F.** Analyte levels in cerebrospinal fluid.

Scatter plots for all four participant groups with median lines added for each group. Values for mean ± 1 SD are given in Table 1. Statistical analysis was performed with pairwise group comparisons of log-transformed analyte levels between controls and AD patients aged ≤62 years (age-adjusted), and between controls and AD patients aged ≥68 years (age-adjusted), as well as between younger and older groups of controls, and younger and older groups of patients with AD. (S1A) t-tau, (S1B) p-tau, (S1C) YKL-40, (S1D) NF-L, (S1E) GFAP, (S1F) progranulin. *Significantly different at the p<0.01 level. **Significantly different at the p<0.001 level. Abbreviations: AD = Alzheimer’s disease, t-tau = total tau, p-tau = phosphorylated tau, NF-L = neurofilament light, GFAP = glial fibrillary acidic protein, EOAD: early-onset AD, LOAD: late-onset AD.

(S1A) (S1B)


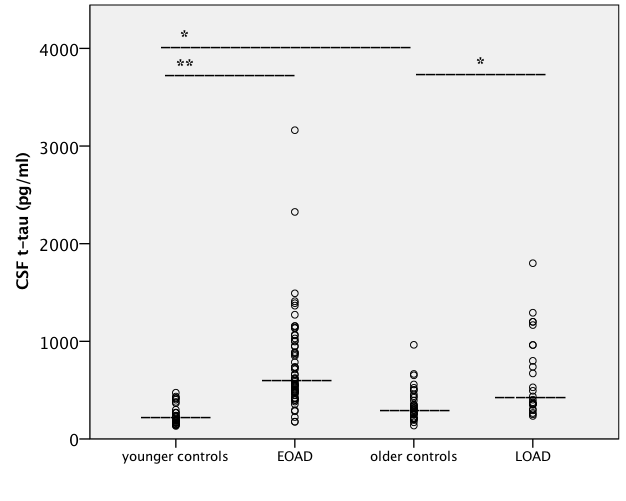

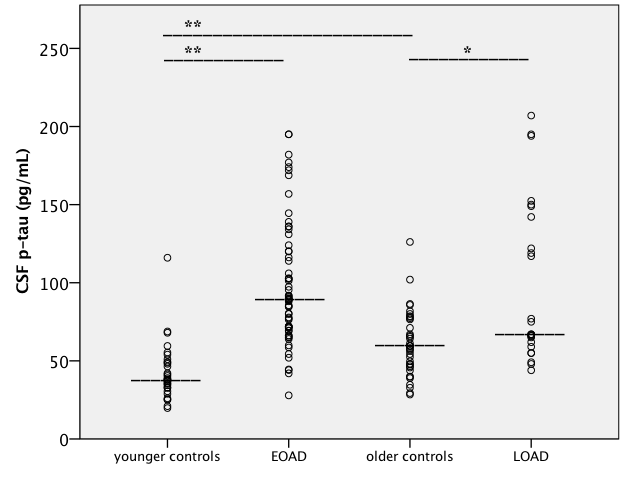


(S1C) (S1D)


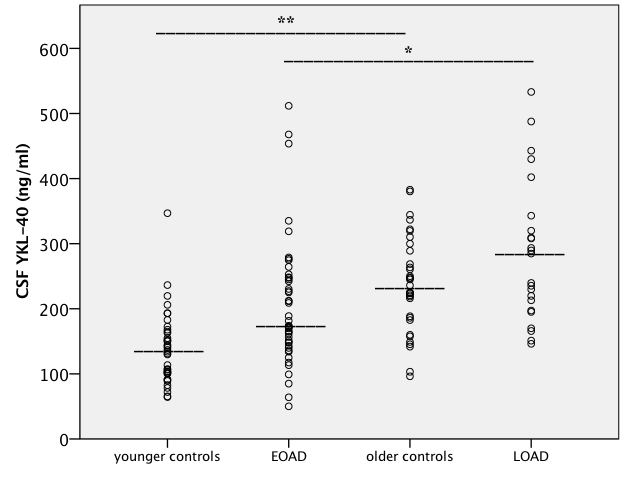

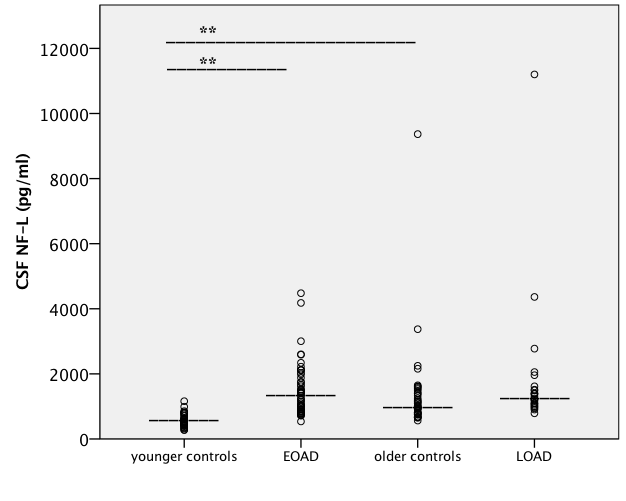


(S1E) (S1F)


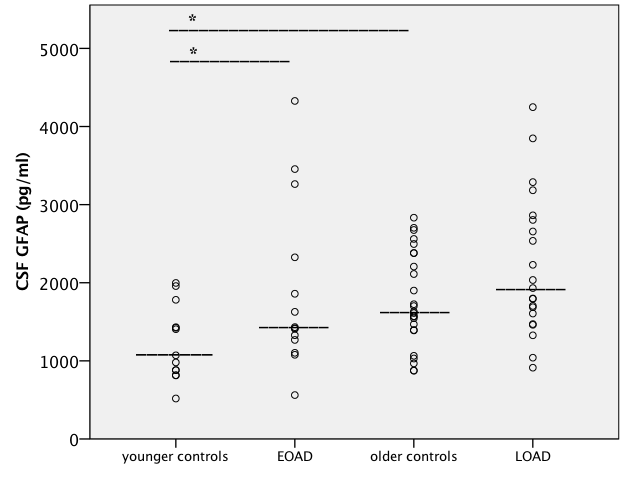

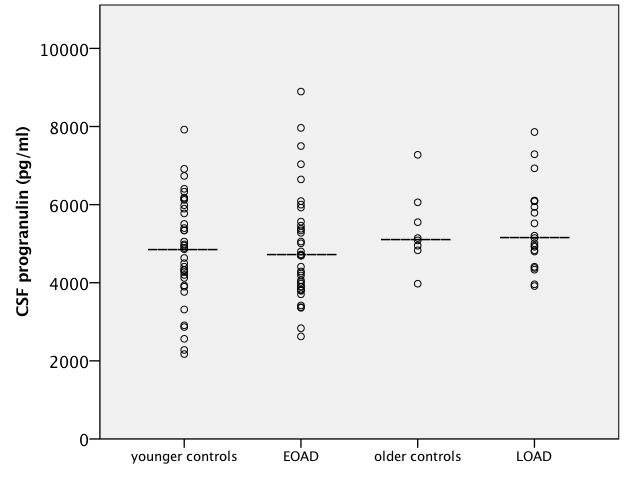

Supplement: Supplementary file 2 [file Data_Sheet_2.docx]
